# Step‐Edge Functionalization by N‐Heterocyclic Carbenes Enhances Catalytic Activity in Electrochemical CO_2_ Reduction

**DOI:** 10.1002/adma.73621

**Published:** 2026-06-09

**Authors:** Philipp Wiesener, Ankita Das, Elena Kolodzeiski, Duong Tran, Ying Pan, Harald Fuchs, Nieves López‐Salas, Saeed Amirjalayer, Frank Glorius, Harry Mönig

**Affiliations:** ^1^ Physical Institute, Center for Nanotechnology (CeNTech) University of Münster Münster Germany; ^2^ Institute of Organic Chemistry University of Münster Münster Germany; ^3^ Department of Chemistry, TUM School of Natural Sciences Technical University of Munich München Germany; ^4^ Sustainable Materials Chemistry University of Paderborn Paderborn Germany; ^5^ Interdisciplinary Center for Scientific Computing Heidelberg University Heidelberg Germany

**Keywords:** atomically defined step‐edges, ${\mathrm{CO}}_{2}$ reduction reaction, electrocatalysis, linear sweep voltammetry, N‐heterocyclic carbenes, non‐contact atomic force microscopy

## Abstract

Atomic step‐edges on metallic surfaces are highly active catalytic sites due to their reduced coordination and modified electronic structure. Yet, approaches to organic ligand functionalization on the single‐molecule level have largely targeted flat terrace geometries, whereas site‐specific step‐edge functionalization remains unaddressed. This study shows that decorating the atomically defined step‐edges of Au(788) with N‐heterocyclic carbenes (NHCs) enhances their catalytic activity toward ${\mathrm{CO}}_{2}$ reduction compared to undecorated metallic step‐edges. Using high‐resolution scanning probe microscopy, an upright‐tilted adsorption geometry and a unified binding mode of three different NHCs at step‐edges are revealed. The exceptional stability of these well‐defined nanostructures allows the use of the single‐crystalline samples as working electrodes in electrochemical experiments. Photoelectron spectroscopy and theoretical simulations correlate charge transfer and conformational details with their catalytic performance. By combining macroscopic electrochemical experiments with single‐molecule microscopy, this study highlights NHC step‐edge functionalization as an effective approach to design highly selective and efficient catalysts.

## Graphical abstract

Atomically precise functionalization by N‐heterocyclic carbenes enhances the catalytic efficiency of macroscopically defined step edges toward electrochemical ${\mathrm{CO}}_{2}$ reduction. Sub‐molecular imaging reveals conformational differences between specific NHCs, which can be correlated with their catalytic performance. Photoelectron spectroscopy and theoretical simulations pinpoint charge transfer and steric effects as major mechanistic parameters.

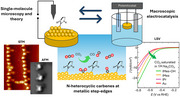

## 1 Introduction

Step‐edges of metallic surfaces play a crucial role in catalysis due to their reduced atomic coordination compared to terrace atoms, resulting in a locally modified electronic structure and lowered reaction barriers [1, 2, 3]. A high density of atomic steps and kinks has been correlated with enhanced catalytic activity, as observed for nanoporous metals [1, 4], nanoparticles [5, 6, 7] and strongly faceted single crystals [8]. Comparative studies between flat and faceted surface terminations reveal that the catalytic performance of the ${\mathrm{CO}}_{2}$ reduction reaction (${\mathrm{CO}}_{2}\mathrm{RR}$) can increase by up to an order of magnitude when step‐edge sites are present [4, 8].

However, in the context of functionalizing metallic surfaces with organic ligands to enhance catalytic properties, the highly active step‐edge sites remain unaddressed. Previous studies have predominantly focused on flat terraces [9, 10, 11, 12, 13, 14, 15, 16] or nanoparticles with various surface geometries [17, 18]. While these approaches significantly improve activity, selectivity, and stability across a broad range of catalytic processes, such as the ${\mathrm{CO}}_{2}\mathrm{RR}$ [16, 18], oxygen dissociation [19], the oxygen reduction reaction (ORR) [20], and the hydrogen evolution reaction (HER) [21], a detailed understanding of site‐specific step‐edge functionalization remains of considerable interest to further develop catalytic performances and to design highly selective catalysts.

In this regard, N‐heterocyclic carbenes (NHCs) are promising and well‐established candidates. Their ability to chemically and electronically functionalize surfaces and nanoparticles offers valuable opportunities to tune catalytic performance [18, 22, 23, 24, 25, 26, 27, 28, 29, 30, 31, 32]. NHCs form strong covalent bonds, exhibit pronounced electron‐donating characteristics, and adopt a variety of structural configurations, making them highly suitable as surface modifiers and molecular anchors [23, 33, 34, 35, 36, 37, 38, 39, 40]. While extensive research has been conducted on NHC adsorption on ideal, flat terraces of metal surfaces [14, 41, 42, 43, 44, 45, 46, 47, 48, 49], their behavior at step‐edges remains largely unexplored. A detailed understanding of the adsorption characteristics and induced electronic surface modifications on the single‐molecule level is essential to fully exploit the potential of NHCs at atomically defined step‐edges.

For NHCs, the actual binding mode between the carbene carbon and the metal substrate strongly influences the reactivity and selectivity for catalytic applications. The binding mode depends on the overall molecular structure and allows the adsorption geometry and electronic properties to be tuned via side‐group modification [15, 50]. Yet, the precise determination of these binding configurations remains a considerable obstacle, as a variety of different binding modes exist. Scanning probe microscopy (SPM) techniques such as scanning tunneling microscopy (STM) and non‐contact atomic force microscopy (nc‐AFM) with functionalized tips have shown to be a promising tool toward elucidating the atomic binding of NHCs on a single‐molecule level. For instance, on flat terraces, the formation of metal‐organic networks composed of flat‐lying NHCs has been reported [14]. Additionally, ordered polymeric structures of mobile and upright‐standing NHCs can be realized through Ullmann coupling [41]. Beyond intermolecular assembly, the substrate itself can stabilize organic molecules on a surface. In particular, atomically defined step‐edges of faceted single crystals can induce molecular ordering over macroscopic length scales [51, 52].

**FIGURE 1:**
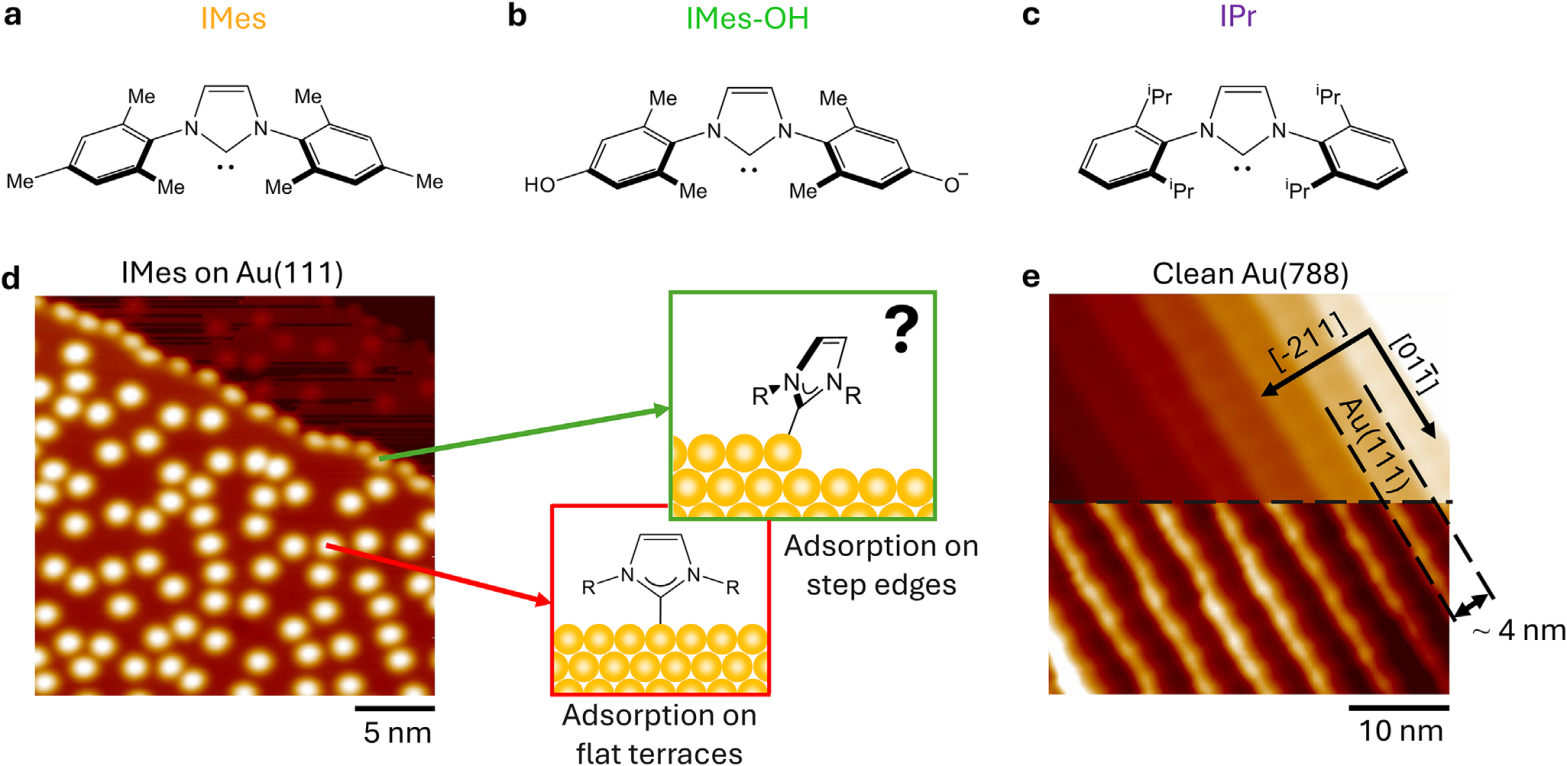
Surface chemistry of NHCs. (a) Structural formula of the used NHC derivatives IMes, 1,3‐dimesitylimidazol‐2‐ylidene; (b) IMes‐OH, 3‐(2,6‐dimethyl‐4‐oxidophenyl)‐1‐(4‐hydroxy‐2,6‐dimethylphenyl)‐1H‐imidazol‐3‐ium‐2‐ide; (c) IPr, 1,3‐bis(2,6‐diisopropylphenyl)imidazol‐2‐ylidene. The IMes‐OH is one‐sided deprotonated, which is confirmed by XPS (Figure S2) and mass‐spectrometry experiments (Figure S3) in the Supporting Information (see also X‐ray diffraction (XRD) measurements in [48]). (d) STM Overview image of IMes deposited on a flat Au(111) surface (feedback 20 pA, 1V). The molecules adsorb on the flat terraces (red frame) and at the step‐edges (green frame). (e) STM Overview image of the clean Au(788) surface (feedback 20 pA, 1V) leveled parallel to the (111) terraces (upper part) and leveled by a linear fit of the scan‐line (lower part).

In this work, we employ nc‐AFM [53] using CuOx‐functionalized tips [54, 55, 56, 57] and STM, complemented by density functional theory (DFT) calculations, to study the nucleation behavior of different NHCs at the atomically defined step‐edges of an faceted Au(788) surface. The NHCs investigated in this study include the well‐known IMes motif (Figure 1a, see also Figure S1a), which is known to adsorb via direct surface bonding in upright geometry on flat terraces. To examine the role of intermolecular interactions in step‐edge nucleation, we also analyze a hydroxyl‐functionalized variant, IMes‐OH (Figure 1b, see also Figure S1b) [48]. The introduction of hydroxyl side groups not only provides an additional pathway for intermolecular interaction via strong OH⋯ $O^{-}$ hydrogen bonds [48], simultaneously it also makes the molecule electron‐richer, which is directly reflected in the tunability of the electronic surface structure. Another NHC motif investigated is IPr (Figure 1c, see also Figure S1c), which preferentially adopts an adatom‐mediated binding configuration, resulting in enhanced surface mobility [42]. In contrast to the distinct binding modes reported for the NHCs on flat terraces, our work reveals a unified binding mode for nucleation at step‐edges.

Furthermore, X‐ray photoelectron spectroscopy (XPS) and electrochemical voltammetry experiments [11, 18] were conducted to correlate conformational properties with their electronic and catalytic properties in the ${\mathrm{CO}}_{2}$ reduction reaction. We show that the catalytic activity of the under‐coordinated step‐edge sites can be further tuned by decorating them with NHCs. A detailed analysis of the charge transfer induced by the NHCs reveals a strong enhancement of charge accumulation along the step‐edge sites. By combining macroscopic experiments with nanoscale microscopy in a single study, detailed insights can be obtained into the relationship between the applied catalysis and the underlying electronic structure of these molecular systems, offering a significant advancement in understanding how their catalytic processes work.

## 2 Results and Discussion

When IMes is deposited onto flat metal terraces (STM image Figure 1d) under ultra‐high vacuum (UHV) conditions, the strong covalent carbene–metal bond leads to a characteristic upright adsorption geometry (Figure 1d, red frame) [41, 42]. In addition, a pronounced nucleation density is observed along the atomic step‐edges (Figure 1d, green frame). Even at submonolayer coverages, the NHCs preferentially saturate these under‐coordinated step‐edge sites, indicating a particularly stable binding mode. Temperature‐dependent desorption experiments (see also Supporting Information, Figure S4) show that NHCs exhibit a higher activation energy for desorption at the step‐edge (1.45 eV ± 0.05) compared to the terrace (1.24 eV ± 0.05). Strong ligand binding is crucial for achieving robust surface functionalization in electrocatalytic applications. In many cases, desorption, particularly under increasingly applied potentials, becomes the limiting factor [33, 34, 35, 58]. Accordingly, NHC functionalized step‐edges provide a promising basis for applications in electrocatalysis.

To bridge the gap between single‐molecule adsorption behavior and the formation of macroscopically defined functional materials, the faceted Au(788) single‐crystal surface with a high density of atomic steps is chosen as a substrate. It corresponds to an Au(111) surface with a macroscopic miscut along the [‐211] direction, consequently, (111) terraces consisting of approximately 16 atomic rows (∼4 nm in width) are formed [52, 59]. An overview STM image of the clean Au(788) surface is shown in Figure 1e. To decorate the step‐edges with NHCs, submonolayer coverages of each compound were deposited by thermal evaporation of the suitable precursor. Subsequently, a mild annealing was performed to desorb excess terrace‐bound NHCs.

### 2.1 NHC‐Nucleation on Atomically Defined Step Edges

**FIGURE 2:**
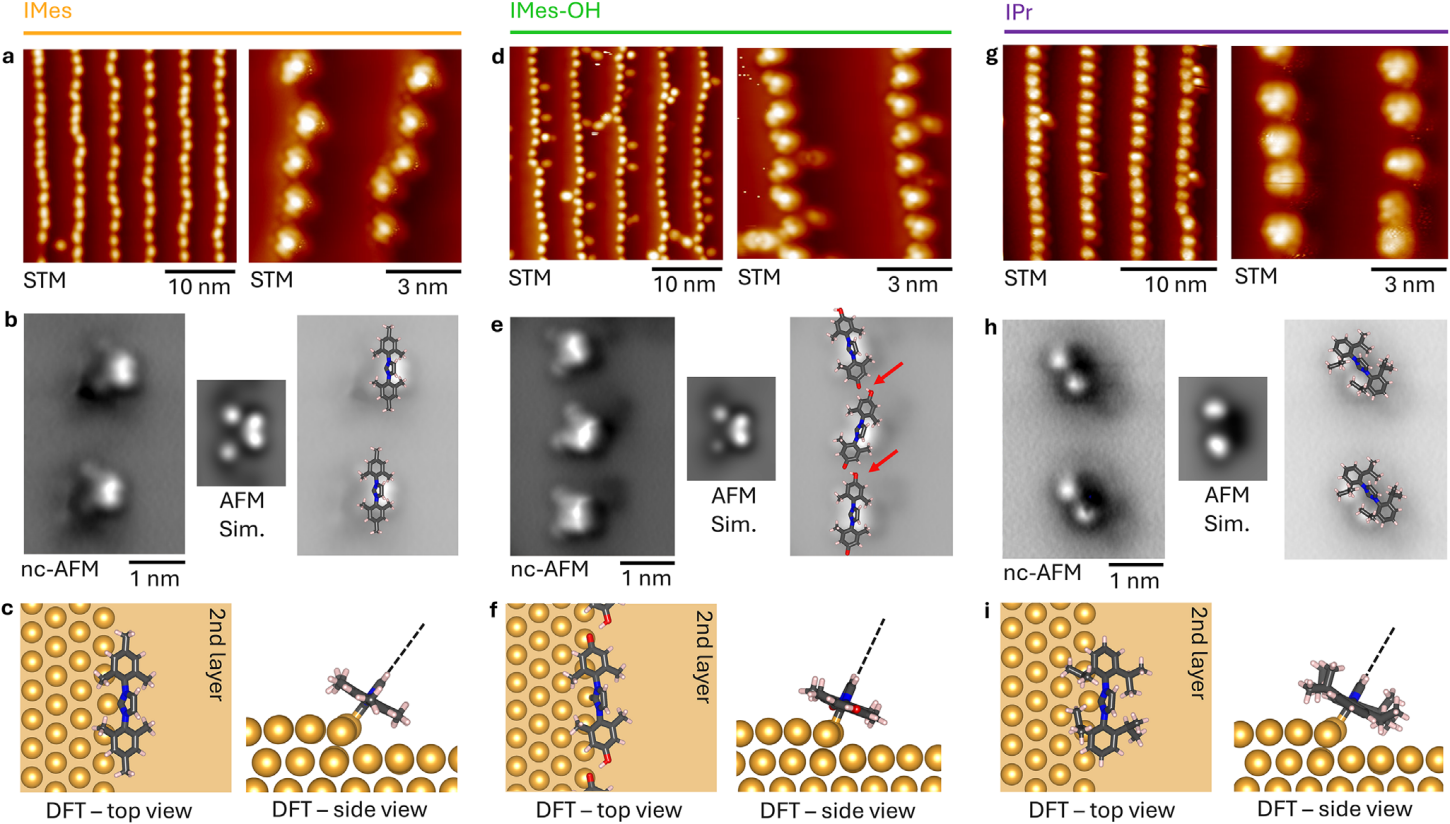
High resolution SPM analysis of NHCs at the step‐edge. Overview (left) and high resolution (right) STM images (feedback 20 pA, 1V) of (a) IMes, (d) IMes‐OH, (g) IPr. Experimental constant height nc‐AFM recorded with a passivated CuOx‐tip (left), AFM simulation (middle) and anticipated structure (right) of (b) IMes, (e) IMes‐OH, (h) IPr. DFT optimized structure as top view and side view for (c) IMes, (f) IMes‐OH, (i) IPr.

An STM image of gold step‐edges fully decorated with IMes is shown in Figure 2a, displaying the NHCs arranged equidistantly along the [01$\bar{1}$] direction and forming a well‐defined nanostructure. For a detailed analysis of the adsorption geometry at the single‐molecule level, constant‐height nc‐AFM with a passivated CuOx‐tip is performed and shown in Figure 2b. The AFM data reveal a repulsive central maximum corresponding to the NHC itself and two additional maxima, attributable to the methyl side groups. This indicates that the molecules are aligned parallel to the step‐edge and adsorbed in an upright configuration. Furthermore, the asymmetric contrast perpendicular to the step‐edge suggests that the molecules are tilted off‐axis. The DFT‐optimized structure derived from the measurements is shown in top and side views in Figure 2c and demonstrates that the molecule is tilted by approximately 36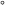 with respect to the surface normal. A DFT‐based AFM simulation in Figure 2b [60] agrees well with the experimental contrast, confirming the molecular tilt. To further quantify the adsorption behavior at the step‐edge, the average molecular distance of IMes is determined to be (1.73 ± 0.08) nm (see also Figure S5) and the molecular density at the step‐edge is calculated to be (0.58 ± 0.08) molecules per nm. Compared to the molecular length of 1.45 nm, the relatively large distance excludes any significant intermolecular interactions and assemblies, as illustrated in Figure 2b.

In contrast, IMes‐OH has been shown to induce molecular ordering through strong hydrogen‐bond interactions, allowing the formation of extended 1D and 2D networks and densely packed assemblies [48]. To investigate how intermolecular interactions influence the nucleation behavior, IMes‐OH functionalized step‐edges are considered (overview STM images in Figure 2d). The corresponding nc‐AFM measurement in Figure 2e reveals a contrast similar to IMes, indicating that the NHCs are aligned parallel to the step‐edge in an upright‐tilted geometry. According to the DFT‐optimized structures shown in Figure 2f the tilt is quantified as 25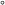 with respect to the surface normal. Notably, the nc‐AFM contrast reveals an additional in‐plane rotation of the NHCs, driven by intermolecular interactions. The OH⋯ $O^{-}$ hydrogen bonds allow the NHCs to arrange more densely along the step‐edge, which is schematically illustrated in Figure 2e (red arrows). Consequently, the molecular density of IMes‐OH at the step‐edge is increased compared to IMes and determined to be (0.72 ± 0.04) molecules per nm (see also Figure S6).

For the catalytic performance of the surface, steric effects, which potentially shield active sites in an electrocatalytic experiment, play a crucial role. To study the effect of more bulky side groups, the adsorption of IPr is investigated. Despite the bulkier isopropyl substituent, the molecules preferentially occupy the step‐edges, as seen by overview STM images in Figure 2g. Again, the AFM data in Figure 2h allow to conclude that the molecules adsorb in an upright configuration with a slight inclination. Due to the molecular tilt, the isopropyl groups, positioned topographically higher, appear as repulsive features, whereas the NHC itself, located 124 pm lower, shows an attractive contrast. Supporting DFT‐optimized structures (Figure 2i) and corresponding AFM simulations (Figure 2h) confirm the adsorption geometry, emphasizing a tilt of 29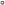 relative to the surface normal. Additionally, an in‐plane rotation of the NHCs is observed within the nc‐AFM images as illustrated in Figure 2h. Presumably, the rotation is primarily attributed to steric effects of the deeper‐lying propyl groups rather than to an assembly by intermolecular interactions. For IPr, the average molecular density at the step‐edge is determined to be (0.78 ± 0.04) molecules per nm (see also Figure S7). The shorter molecular backbone of IPr enables significantly closer packing along the step‐edge compared to IMes and IMes‐OH.

Overall, the presented data provide a detailed structural analysis and insight into the adsorption geometries of the three investigated NHCs at gold step‐edges on the single‐molecule level. The NHCs bind to the metal substrate in an upright configuration, exhibiting both in‐plane rotation and tilt relative to the surface normal. Furthermore, to investigate the binding mode of the NHCs for atomically defined step‐edges, a detailed STM and DFT analysis is presented in the Supporting Information (Figure S8). Elucidating the binding mode, such as direct surface bonding or adatom bonding, is essential for understanding NHC nucleation and corresponding catalytic processes [15]. On atomically flat terraces, the investigated NHCs exhibit non‐uniform binding modes. For instance, IMes preferentially forms direct surface bonds, whereas IPr adopts a mobile ‘ballbot’ configuration through adatom bonding [42]. IMes–OH, in contrast, has been shown to form extended hydrogen‐bonding networks that incorporate both binding modes [48]. Remarkably, the analysis reveals that for all NHCs studied, the carbene binds directly to a step‐edge gold atom rather than via a mediating adatom. This is also reflected in the more stable binding configuration at the step‐edges compared to more diffuse species formed on flat terraces, which is, in regard to surface stability, inefficient.

### 2.2 Benchmarking the Electrocatalytic Activity Toward ${\mathrm{CO}}_{2}$ Reduction

**FIGURE 3:**
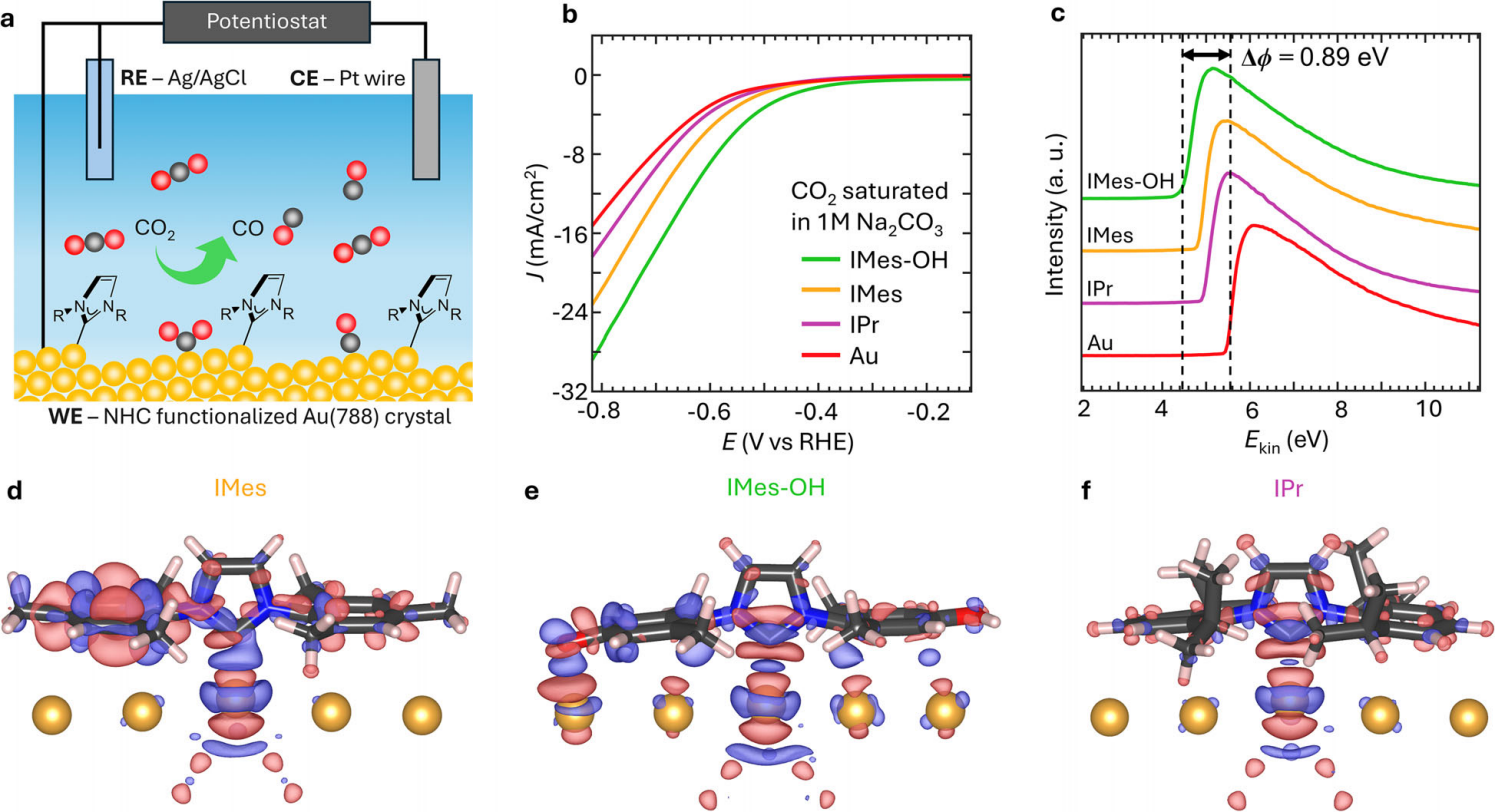
Catalytic performance and electronic modification of the NHC‐functionalized surfaces. (a) Schematics of the electrochemical cell. Reference electrode (RE), counter electrode (CE), working electrode (WE). (b) Linear sweep voltammetry curves for the electrochemical reduction of ${\mathrm{CO}}_{2}$ to CO. (c) Secondary electron cutoff using XPS. For better visibility the curves are shifted vertically. (d–f) Charge difference plots (negative in blue, positive in red, iso‐level 1.6 me/Å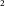) calculated out of the DFT optimized structures in Figure 2 for (d) IMes, (e) IMes‐OH, (f) IPr. For better visibility only the molecule and the step‐edge atoms are shown.

With the high‐resolution SPM data and derived molecular geometries in hand, the catalytic and electronic properties of these well‐defined nanostructures can be systematically explored. Therefore, the NHC functionalized single crystals were removed from the UHV environment and transferred into a custom‐made electrochemical cell under ambient conditions with ${\mathrm{CO}}_{2}$‐saturated aqueous 1 M ${\mathrm{Na}}_{2}$ ${\mathrm{CO}}_{3}$ as electrolyte. Subsequently, the electrochemical reduction of ${\mathrm{CO}}_{2}$, catalyzed by the NHC‐functionalized surfaces, was evaluated by linear sweep voltammetry (LSV). The schematics of the experiment is shown in Figure 3a, while a more detailed description of the electrochemical setup is provided in the Supporting Information (Figure S9). All reported potentials are referenced to the reversible hydrogen electrode (RHE).

Figure 3b presents the LSV curves of the IMes‐, IMes‐OH‐, and IPr‐functionalized Au(788) surfaces, along with the clean, unmodified Au(788) crystal as a reference. The NHC‐functionalized surfaces exhibit increased total current densities and positively shifted onset potentials compared to bare Au(788), indicating enhanced catalytic activity induced by the NHC modification. In contrast, significantly lower current densities and a negatively shifted onset potential were observed when the samples were tested in $N_{2}$‐saturated aqueous ${\mathrm{Na}}_{2}$ ${\mathrm{CO}}_{3}$ electrolyte (see also Figure S10), emphasizing that the enhanced activity of the NHC‐functionalized surfaces originates from ${\mathrm{CO}}_{2}$ reduction. In good agreement, Cao et al. [18] demonstrated that NHC surface functionalization of gold nanoparticles selectively enhances ${\mathrm{CO}}_{2}$ reduction activity in preference to the hydrogen evolution reaction. Within our data, a clear trend among the different NHC species can be observed. The hydroxyl‐functionalized NHC, IMes‐OH, shows the highest total current density and the most positive onset potential of ‐0.56 V versus RHE for the ${\mathrm{CO}}_{2}$ reduction, followed by the IMes functionalized surface of ‐0.61 V versus RHE. The IPr‐functionalized surface displays the most negative onset potential of ‐0.64 V versus RHE among the three, while the bare Au(788) surface exhibits an onset potential of ‐0.67 V versus RHE. The onset potentials were determined at the point where a total current density of 2 mA ${\mathrm{cm}}^{-2}$ was first reached.

### 2.3 NHC‐Induced Modification of the Electronic Surface Structure

A key aspect in understanding the catalytic performance of NHCs on metal surfaces lies in their electron‐donating ability via the strong covalent carbene–metal bond. To quantify corresponding electronic surface modifications, the work function ϕ of the NHC functionalized surfaces, measured as the secondary electron cutoff using XPS and shown in Figure 3c, is calculated. In agreement with literature [61], the NHC‐functionalized surfaces exhibit a reduced work function compared to the clean Au(788) substrate. Analogous to the observed trend in catalyzing the ${\mathrm{CO}}_{2}$ reduction, the reduction of the work‐function follows the same order. IMes‐OH induces the strongest reduction, lowering the work function to 4.68 eV, followed by IMes (4.96 eV) and IPr (5.09 eV). The work function of the pristine Au(788) surface was determined to be 5.57 eV.

These findings demonstrate not only a strong consistency between the UHV and electrochemical measurements. Furthermore, a clear correlation between the reduction of the work function and catalytic activity is revealed, which establishes an ideal basis for a more detailed analysis of the active sites. The high‐resolution SPM data and DFT‐derived geometries, presented in the previous section, enable a precise theoretical analysis of the charge transfer between the molecule and the gold surface, which allows to consider the modification of the electronic structure at the interface in more detail. The calculated charge difference plots for the three investigated NHCs are shown in Figure 3d–f.

In all cases, the covalent carbene–metal bond enables charge redistribution between molecule and substrate, which can be identified by a strong contrast in the charge difference. Our data indicate that the inherently active Au step‐edges are decisive for the ${\mathrm{CO}}_{2}$ reduction reaction [8], while the NHCs activate metal sites in their vicinity through distinct electron donation. Although step‐edge sites are partially blocked for ${\mathrm{CO}}_{2}$ adsorption by the NHCs, their presence modify the local charge distribution and further reduce reaction barriers in the step‐edge region. For IMes‐OH, an additional charge‐transfer channel arises via the deprotonated oxygen atom and the hydroxyl side group. The functionalization of the NHC backbone with this electron‐rich substituent facilitates the overall electron accumulation near the step‐edge, leading to a pronounced reduction of the work function and increase of the catalytic activity.

**FIGURE 4:**
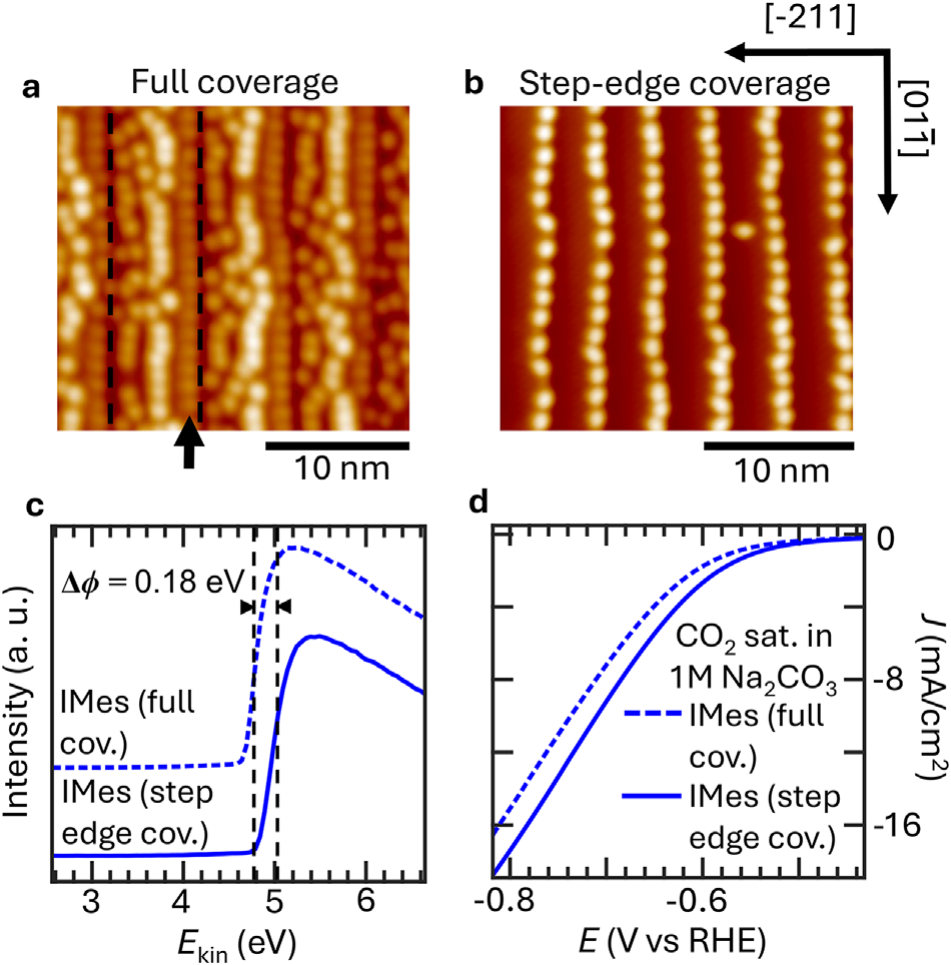
Coverage dependence of the catalytic performance and reduction of the work function for IMes. (a) Overview STM image (feedback 20 pA, 1 V) of Au(788) with full coverage of IMes, leveled by a linear fit of the scan‐line. A single (111) terrace is highlighted by two black dashed lines. The NHCs located at the step edges (indicated by the black arrow) exhibit a slightly darker contrast, which can be attributed to their tilted adsorption geometry compared to the more upright‐oriented NHCs on the terraces. (b) Overview STM image (feedback 20 pA, 1 V) of Au(788) with only the step‐edges functionalized. (c) Secondary electron cutoff using XPS. For better visibility the curves are shifted vertically. (d) Linear sweep voltammetry curves for the electrochemical reduction of ${\mathrm{CO}}_{2}$ to CO. The blue dashed lines correspond to measurements of a fully covered surface (STM in Figure 4a), whereas the blue solid lines correspond to a surface where only the step edges are functionalized (STM in Figure 4b). See also Figure S11, which includes the corresponding curves for bare Au(788).

To investigate the role of ${\mathrm{CO}}_{2}$ accessibility to the active step‐edge vicinity, a direct comparison between a fully covered Au(788) surface (STM image in Figure 4a) and a step‐edge‐functionalized surface (STM image in Figure 4b) is evaluated for IMes. The corresponding secondary electron cut‐off data (Figure 4b) show that the reduction in work function, and thus the NHC‐induced charge transfer, is increased by 0.18 eV for a fully covered surface compared to a surface where only the step‐edges are functionalized. This observation is consistent with previous studies, which demonstrate that the work function reduction induced by NHCs increases with surface coverage [37, 61]. Notably, despite the enhanced electronic modification, the LSV curve (Figure 4c) of the fully covered surface exhibits lower total current densities and a negatively shifted onset potential by 24 mV compared to the selectively functionalized step‐edge system. This reduced catalytic performance can be attributed to the limited steric accessibility of ${\mathrm{CO}}_{2}$ to the underlying gold step‐edge region. At full coverage, the NHC layer partially blocks the interaction between ${\mathrm{CO}}_{2}$ and the metal, thereby suppressing the overall ${\mathrm{CO}}_{2}$ reduction reaction.

Focusing on the selectively step‐edge functionalized systems, the numerical values obtained from experiment and theory are summarized in Table 1. The correlation between the catalytic performance and the work function ϕ emphasizes the NHCs important role in tuning electronic surface properties. In contrast, the uncorrelated molecular densities D along the step‐edge underscore the fact, that not only the spatial density of NHCs on a surface matters. As seen for IPr and the fully covered IMes surface, steric effects, can shield surrounding active sites, leading to a reduced catalytic performance. Furthermore, the binding energy plays an important role in achieving a stable structure and a robust catalyst. The experimental activation energy for desorption $E_{\text{desorb,exp}}$ calculated out of the temperature‐dependent desorption experiments and the theoretical binding energy $E_{\text{bind,theo}}$ calculated out of the DFT‐optimized structures is presented (for details, see also Supporting Information). The intermolecular hydrogen bonding and strong interaction between the substrate and side groups of the IMes‐OH support the bonding at the step‐edges and makes this NHC a more promising molecular anchor compared to IMes. In contrast, IPr exhibits the lowest binding energy, which is consistent with its comparatively weak catalytic performance.

**TABLE 1:** Numerical values obtained from experiment and theory. Work function ϕ extracted at the FWHM of the secondary electron cutoff using XPS. Onset potential E extracted at the point where a total current density of 2 mA ${\mathrm{cm}}^{-2}$ was first reached in the LSV. Average molecular density D along the step‐edge determined with STM. Experimental activation energy for desorption $E_{\text{desorb,exp}}$ and theoretical binding energy $E_{\text{bind,theo}}$ (see also Supporting Information).

Molecule	E (V vs. RHE)	ϕ (eV)	$E_{\text{desorb,exp}}$ (eV)	$E_{\text{bind,theo}}$ (eV)	D (nm)
IMes‐OH	−0.56	4.68	1.49 ± 0.05	4.43	1.39 ± 0.04
IMes	−0.61	4.96	1.45 ± 0.05	3.52	1.73 ± 0.08
IPr	−0.64	5.09	1.37 ± 0.05	3.30	1.27 ± 0.04
Au	−0.67	5.57	—	—	—

## 3 Conclusion

By correlating sub‐molecular imaging techniques with voltammetry, we demonstrate the formation of a macroscopically defined and catalytically active nanostructure for the ${\mathrm{CO}}_{2}$ reduction reaction by decorating the atomically defined step‐edges of the Au(788) surface with three different NHCs. In all cases, an upright‐tilted adsorption geometry is observed and, in contrast to flat terraces, a unified binding mode is identified. In particular, the carbene binds directly to a step‐edge gold atom rather than via an adatom. Consistently, we demonstrate that NHC nucleation is significantly more stable at the step edges than on flat terraces. The molecular side groups play a crucial role, not only in governing intermolecular interactions but also in defining the electronic structure of the molecule, which directly influences the capability of tuning the surface properties. As a result, NHC functionalized step‐edges become more electron‐rich, leading to a reduction of the work function and thereby facilitating catalytic reactions. Our findings spotlight the important role and opportunities of step‐edge functionalization by NHC compounds to design highly efficient and selective catalysts with defined active sites.

## 4 Experimental Section

### 4.1 Synthesis

For the synthesis of IMes‐OH (3‐(2,6‐dimethyl‐4‐oxidophenyl)‐1‐(4‐hydroxy‐2,6‐dimethylphenyl)‐1H‐imidazol‐3‐ium‐2‐ide), we refer to Liu et al. [48]. For the synthesis of IMes (1,3‐dimesitylimidazol‐2‐ylidene) and IPr (1,3‐bis(2,6‐diisopropylphenyl)imidazol‐2‐ylidene) we refer to Wang et al. [42]. Details on the adducts of the NHCs, as well as related XPS‐, and mass spectrometry measurements of the compounds, are available in the Supporting Information.

### 4.2 SPM and XPS Measurements

The used setup consisted of a scanning probe microscopy (SPM) system and a X‐ray photoelectron spectroscopy (XPS) system, which were connected in UHV to enable an in situ vacuum transfer. The low‐temperature SPM with the MATRIX SPM control system (LT‐STM/AFM) from Scienta Omicron was operated under ultra‐high vacuum conditions with a base pressure below 5 x 10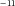 mbar and cooled to 78 K by a liquid nitrogen bath cryostat. The used qPlus force sensors [53] enabled STM and AFM data recording with resonance frequencies $f_{0}$ of 22–28 kHz and quality factors of 5–15k. The AFM experiments were performed with a constant amplitude (1.0 Å) active feedback loop and in constant height mode with a bias voltage of 0 V and with a CuOx‐tip. The STM images were recorded with a metal tip and a constant current active feedback loop. To prevent tip changes, low tunneling current setpoints of 20 pA and bias voltages of 1 V were used. Raw image data were processed with a Gaussian filter (Scanning Probe Image Processor, SPIP 5.1). The XPS system from Specs was used with an Al–Kα X‐ray source, a hemispherical analyzer (Specs Phoibos 100) and a 2D delay line detector and was operated at a base pressure below 5 x 10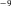 mbar. For measuring the secondary electron cut‐off, a voltage of 8.0 V was applied to the sample and later subtracted from the measurement.

### 4.3 AFM Tip Functionalization

To form a CuOx‐tip, chemically and focused ion beam (FIB) etched tungsten tips were indented in the partially oxidized Cu(110)O(2x1)‐surface, as described in literature [57]. Before recording all of the shown AFM data, a constant height nc‐AFM measurement of an oxide stripe on the Cu(110)O(2x1)‐surface was taken to confirm the covalent tetrahedral bonding configuration of the terminal oxygen and typical imaging contrast [56, 62, 63].

### 4.4 Sample Preparation

For the sample preparation, the Au(788) single crystal was cleaned by cycles of Ar+ sputtering and subsequent annealing up to 600 K, each for 10 min, before the NHCs were deposited by thermal evaporation. Therefore, the molecular powder of IMes, IPr, and IMes‐OH was heated up to 338, 348, and 573 K, respectively. The single crystal at room temperature was placed in the flux for 5–10 min. Finally, a subsequent mild annealing to 510–540 K was performed to desorb excess terrace‐bound NHCs (see also Figure S4). The temperatures were measured with a pyrometer.

### 4.5 Electrochemical Experiments

All the experiments were carried out in a custom‐made three‐electrode electrochemical cell (see also Figure S9) under ambient conditions. The Au(788) single crystal (⌀9 mm with an ⌀8 mm area exposed to the electrolyte) was used as the working electrode. For the measurements shown in Figure 4, a different crystal was used than for those in Figure 3. The polishing conditions from crystal to crystal, and consequently the density of step edges, may differ on a macroscopic scale. The data were largely comparable, however, the overall total current densities show slight variations. For comparison, see also Figure S11. The crystal was mounted in the voltammetry sample holder and connected to a metallic current collector. A coiled platinum wire was used as a counter electrode and Ag/AgCl (in sat. KCl) as a reference electrode. The electrolyte (1 M ${\mathrm{Na}}_{2}$ ${\mathrm{CO}}_{3}$) was saturated with ${\mathrm{CO}}_{2}$ (pH 9, tested by a pH stripe) for at least 20 min before the measurement. Initially, cyclic voltammetry from 0.1 to ‐0.8 V versus Ag/AgCl was conducted until the setup was stabilized. The ${\mathrm{CO}}_{2}$ reduction reaction was evaluated by means of linear sweep voltammetry at a scan rate of 50 mV/s from 0.1 to ‐1.50 V versus Ag/AgCl. Three sweeps were recorded each time. All reported potentials were measured against the Ag/AgCl reference electrode and converted to the RHE reference scale by E(RHE) = E(Ag/AgCl) + 0.197 V + 0.0591 × pH.

### 4.6 Computational Details

Utilizing the Vienna Ab Initio Simulation Package (VASP) for periodic density functional theory (DFT) calculations [64, 65], atomistic investigations were conducted on the Au surfaces focusing on geometry optimization, the local potential (LOCPOT) and the charge density (CHGCAR). In all calculations the optPBE‐vdw functional [66] was employed, together with projected augmented wave (PAW) pseudo‐potentials and a plane‐wave cutoff of 600 eV for the wave functions, was employed. A Fermi smearing with a standard deviation of 0.2 eV described the electronic states. Monkhorst Pack k‐point sampling with an 1×1×1 mesh was employed for Brillouin zone integration in all calculations. Convergence tests comparing different k‐point meshes in Figure S12 show that the calculated binding energy exhibits only minor sensitivity to k‐point sampling, indicating that it is already well converged at a 1x1x1 grid. The slab models of the monomers (IMes and IPr) consist of three layers of an aperiodic Au(111) 8x8 unit cell and a fourth 4x8 layer on top to form the step‐edge. The unit cell used for modeling the self‐assembly (IMes‐OH) considers three layers of a periodic Au(111) 5x10 unit cell and a fourth 5x5 layer. The geometries including the molecules were fully optimized, keeping only the bottom layer constant of 3.64 Å. For all calculations, a dipole correction to the energy for the slab model was carried out perpendicular to the surface. The convergence criteria for the forces on the nuclei were 0.01 eV Å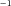 for the geometry optimization and $10^{-8}$ eV for the electronic relaxation. Charge density differences are computed as $\Delta \rho =\rho_{\text{total}}-\rho_{\text{slab}}-\rho_{\text{molecule}}$, where all densities were evaluated using the same atomic geometries.

### 4.7 AFM and STM Simulations

For AFM simulations, the probe particle model by Hapala et al. was used [60]. To generate the electrostatic force field and the Lennard‐Jones force field within this model, the DFT optimized structures and the calculated local potential were used. The simulated relaxed scan was then performed by using an oxygen probe particle with a charge of ‐0.95e [56], a lateral spring constant of 161.9 N/m, a radial spring constant of 271.1 N/m and a equilibrium position of (0.0 0.0 1.8) Å [62]. The STM simulations were calculated based on the Tersoff‐Hamann approximation [67]. For these calculations, the convergence threshold for electronic relaxation is set to 10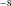.

## Funding

This work was funded by the Deutsche Forschungsgemeinschaft (DFG, German Research Foundation) through projects 519972808, SFB 1459 and SFB 1249 (project C14).

## Conflicts of Interest

All authors declare no financial/commercial conflicts of interest.

## Supporting information


**Supporting File**: adma73621‐sup‐0001‐SuppMat.pdf.

## Data Availability

The data shown in this manuscript are available from the authors upon reasonable request.
