# Supplementary material for: Step‐Edge Functionalization by N‐Heterocyclic Carbenes Enhances Catalytic Activity in Electrochemical CO2 Reduction
Source: Adv Mater. 2026 Jun 9;38(39):e73621. doi: 10.1002/adma.73621 (PMC13361271; doi:10.1002/adma.73621)
Supplement: Supplementary file 1 — Supporting File: adma73621‐sup‐0001‐SuppMat.pdf. [file ADMA-38-e73621-s001.pdf]

# Supporting Information - Step-Edge Functionalization by N-Heterocyclic Carbenes Enhances Catalytic Activity in Electrochemical $CO_2$ Reduction

Philipp Wiesener[1], Ankita Das[2], Elena Kolodzeiski[3], Duong Tran[1], Ying Pan[4], Harald Fuchs[1], Nieves López-Salas[4*], Saeed Amirjalayer[5*], Frank Glorius[2*], Harry Mönig[1*]

[1]Physical Institute, Center for Nanotechnology (CeNTech), University of Münster, Wilhelm-Klemm-Straße 10, 48149 Münster, Germany.
[2]Institute of Organic Chemistry, University of Münster, Corrensstraße 36, 48149 Münster, Germany.
[3]Department of Chemistry, TUM School of Natural Sciences, Technical University of Munich, Lichtenbergstraße 4, 85748 Garching bei München, Germany.
[4]Sustainable Materials Chemistry, University of Paderborn, Warburger Straße 100, 33098 Paderborn, Germany.
[5]Interdisciplinary Center for Scientific Computing, University of Heidelberg, Berliner Straße 43, 69120 Heidelberg, Germany.

*Corresponding authors. E-mails: nieves.lopez.salas@uni-paderborn.de; saeed.amirjalayer@iwr.uni-heidelberg.de; glorius@uni-muenster.de; harry.moenig@uni-muenster.de;

# Calculations for determining the binding energies

The binding energies of the NHCs were determined both experimentally and theoretically. First, temperature-dependent desorption experiments were carried out to observe desorption from flat terraces and step edges. A full monolayer of each NHC was deposited on the Au(788) surface, followed by annealing at stepwise increasing temperatures. After each annealing step, the sample surface was imaged using STM to determine the remaining coverage on flat terraces and at step edges (Fig. S4). The measurements show that adsorption at the step edges is more stable compared to adsorption on the terraces. IMes-OH exhibits generally increased desorption temperatures due to additional intermolecular hydrogen bonding.

To quantify the desorption behavior, the experimental activation energy for desorption $E_{\mathrm{desorb,exp}}$ was calculated from the data using the Redhead equation:

$$E_{\mathrm{desorb,exp}} = RT\left[\ln\left(\frac{\nu T}{\beta}\right) - 3.64\right], \tag{1}$$

where $R$ is the ideal gas constant, $T$ is the desorption temperature, $\beta = 1\ \mathrm{Ks}^{-1}$ is the heating rate, and $\nu = 10^{-13}\ \mathrm{s}^{-1}$ is the pre-exponential rate constant [1]. The desorption temperature $T$ [K] was defined as the temperature at which no occupancy at the step edges/terraces was observed in the STM contrast. The theoretical binding energy $E_{\mathrm{bind,theo}}$ was calculated from the DFT-optimized structures. It should be noted that activation energies derived from temperature-dependent desorption using the Redhead equation are not directly equivalent to binding energies obtained from DFT calculations. The experimental values correspond to the energy barrier that must be overcome for the adsorbate to leave the surface. This approach relies on simplifying assumptions, including a fixed pre-exponential factor and idealized desorption kinetics. In contrast, DFT provides a 0 K adsorption energy. Even for structurally well-defined and defect-free surfaces, deviations between experimental and theoretical values are therefore expected. These differences arise from entropic and finite-temperature contributions inherent to the experimental measurement, uncertainties associated with the assumed pre-exponential factor in the Redhead analysis, and the approximations of the employed exchange–correlation functional within DFT. While absolute values may differ, the relative trends in adsorption energies and activation energies are typically more robust and in the present case consistent between experiment and theory.

# STM and DFT analysis regarding ad-atom mediated binding

In Fig. S8, a detailed STM and DFT analysis is presented to show that the carbene is directly bonded to a step-edge gold atom (Fig. S8b) rather than through a mediating metal ad-atom (Fig. S8a). First, STM simulations were calculated from the DFT-optimized structures for both cases (Fig. S8c-e, bottom) and then compared to the corresponding experimental STM data (Fig. S8c-e, top). For each NHC, line profiles corresponding to the ad-atom binding (blue) and direct binding (orange) were extracted and are shown in Fig. S8c-e (middle). As a reference, an additional line profile of the unoccupied step edge (yellow) was taken and used to horizontally align the curves. The analysis clearly shows for all NHCs, that the carbene is directly bonded to a step-edge gold atom rather than through a mediating metal ad-atom.

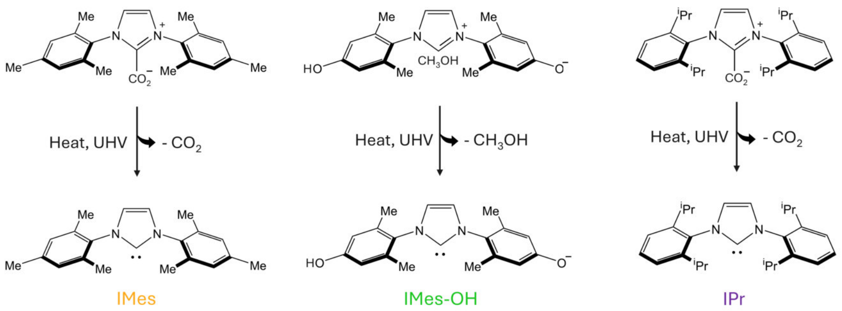


**Fig. S1 Structural formulas and details on the adducts of the NHCs.** The NHCs are synthesized with an adduct, which protects the carbene upon metalization on the surface. IMes and IPr are synthesized with a $CO_2$ adduct and IMes-OH with a $CH_3OH$ adduct. The IMes-OH is one-sided deprotonated, which is confirmed by XPS (Figure S2), mass-spectrometry experiments (Figure S3) and X-ray diffraction (XRD) measurements in Ref. [2]).

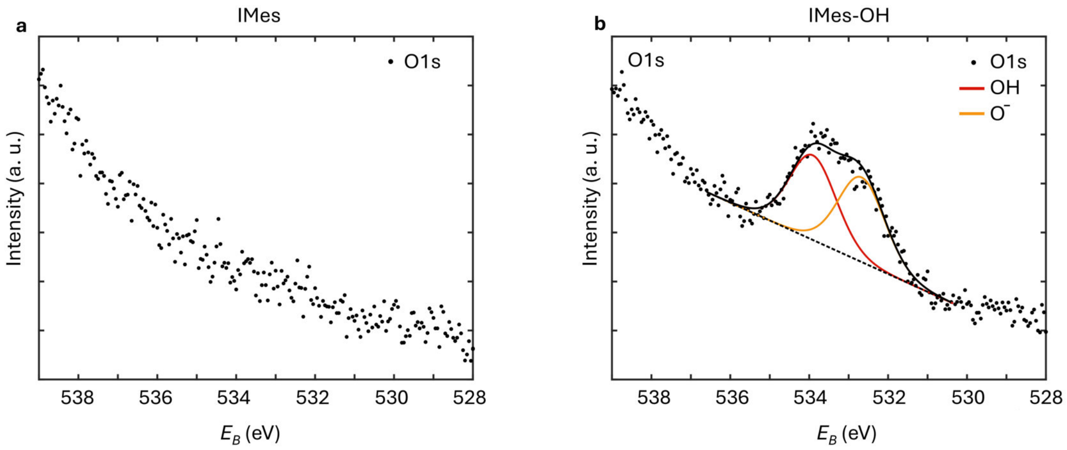


**Fig. S2 XPS analysis of IMes and IMes-OH.** O 1s XPS spectrum of a monolayer of **(a)** IMes and **(b)** IMes-OH on Au(111). For IMes, it shows a negligible trace of oxygen, while for IMes-OH two peaks can be clearly identified, corresponding to the OH-side group and the deprotonated oxygen.

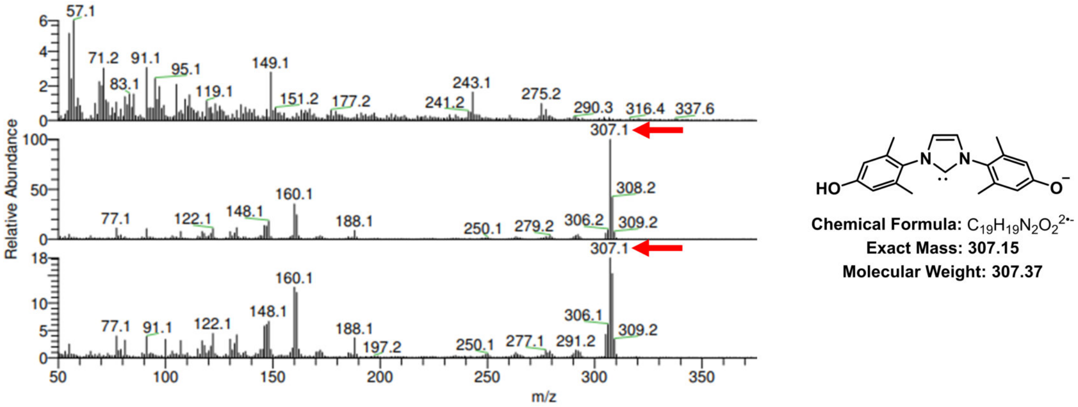


**Fig. S3** Direct-inlet mass spectrometry of the IMes-OH NHC precursor [2].

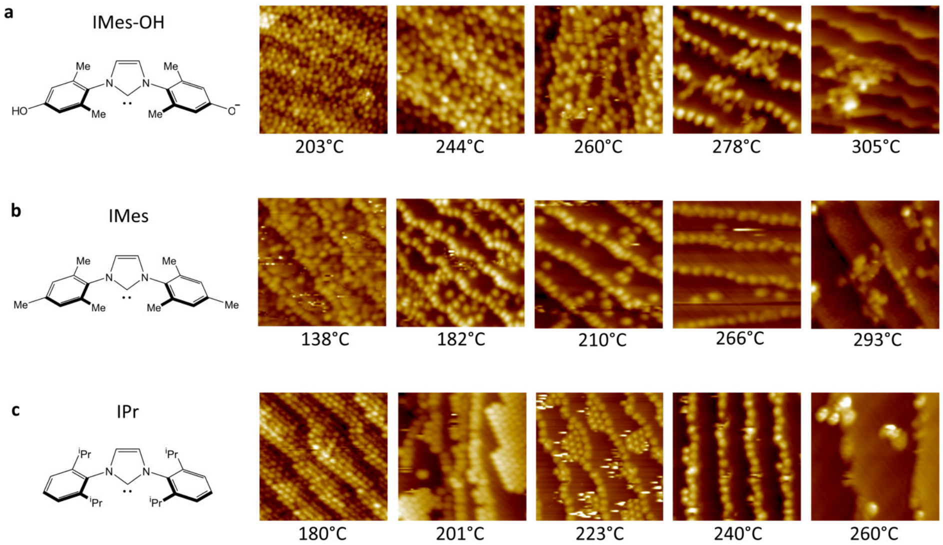


**Fig. S4 Temperature-dependent desorption experiments using STM.** A full monolayer of **(a)** IMes-OH, **(b)** IMes and **(c)** IPr was deposited on the Au(788) surface. Subsequently, an annealing with stepwise increasing temperature was performed to observe the desorption on flat terraces and step edges.

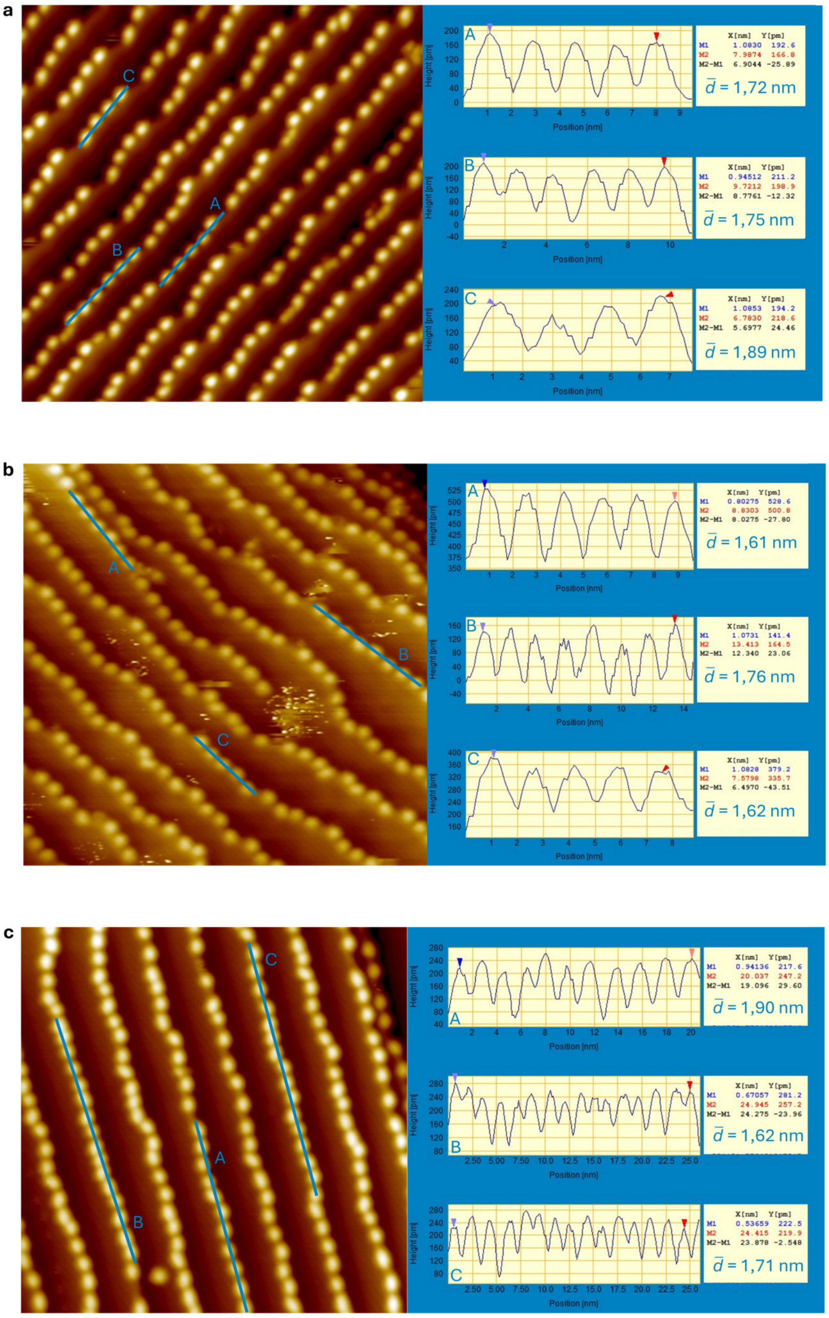


**Fig. S5 STM images for determining the average intermolecular distance of IMes.** For three different STM experiments (feedback 20 pA, 1 V) in **(a)**-**(c)** the intermolecular distance is determined. Within each measurement, three line scans along the NHC-functionalized step edge are evaluated.

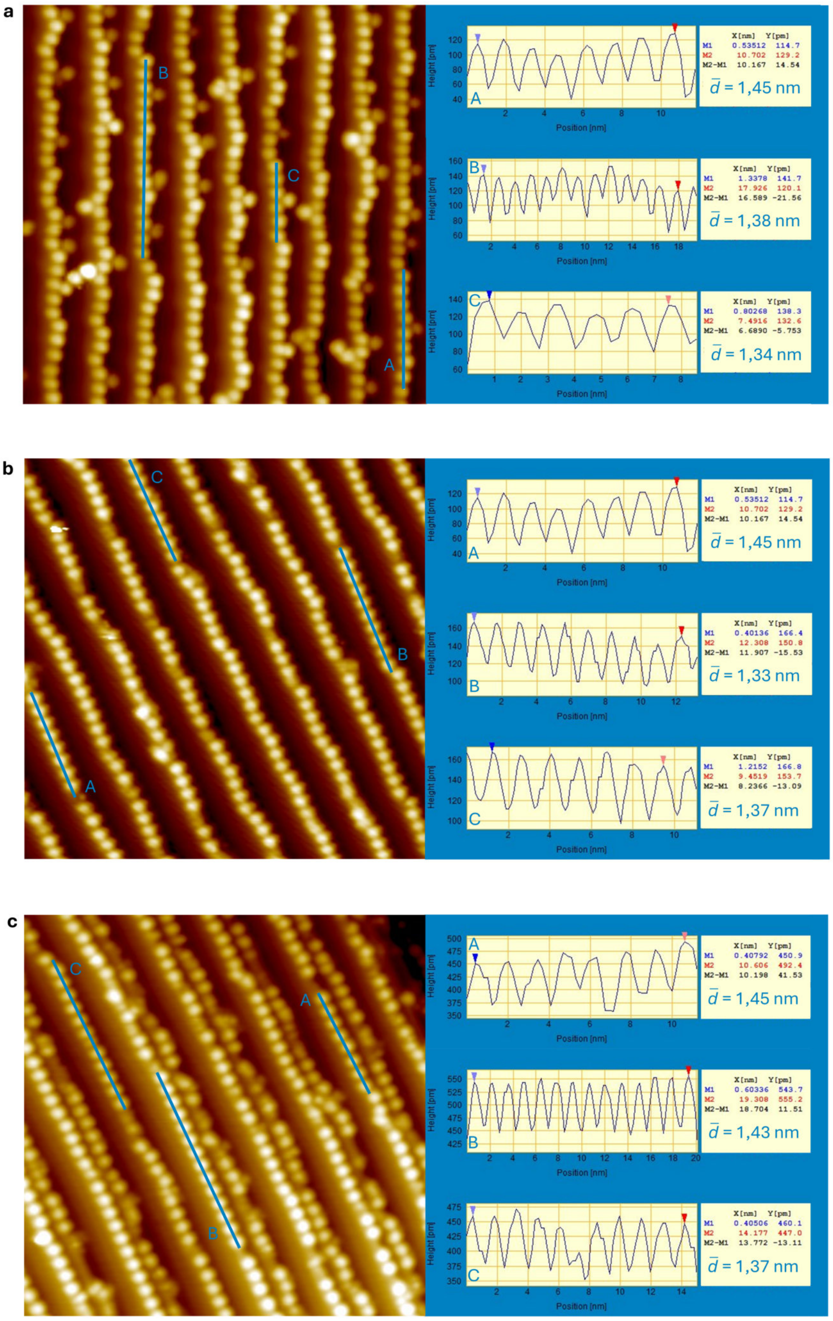


**Fig. S6 STM images for determining the average intermolecular distance of IMes-OH.** For three different STM experiments (feedback 20 pA, 1 V) in **(a)**-**(c)** the intermolecular distance is determined. Within each measurement, three line scans along the NHC-functionalized step edge are evaluated.

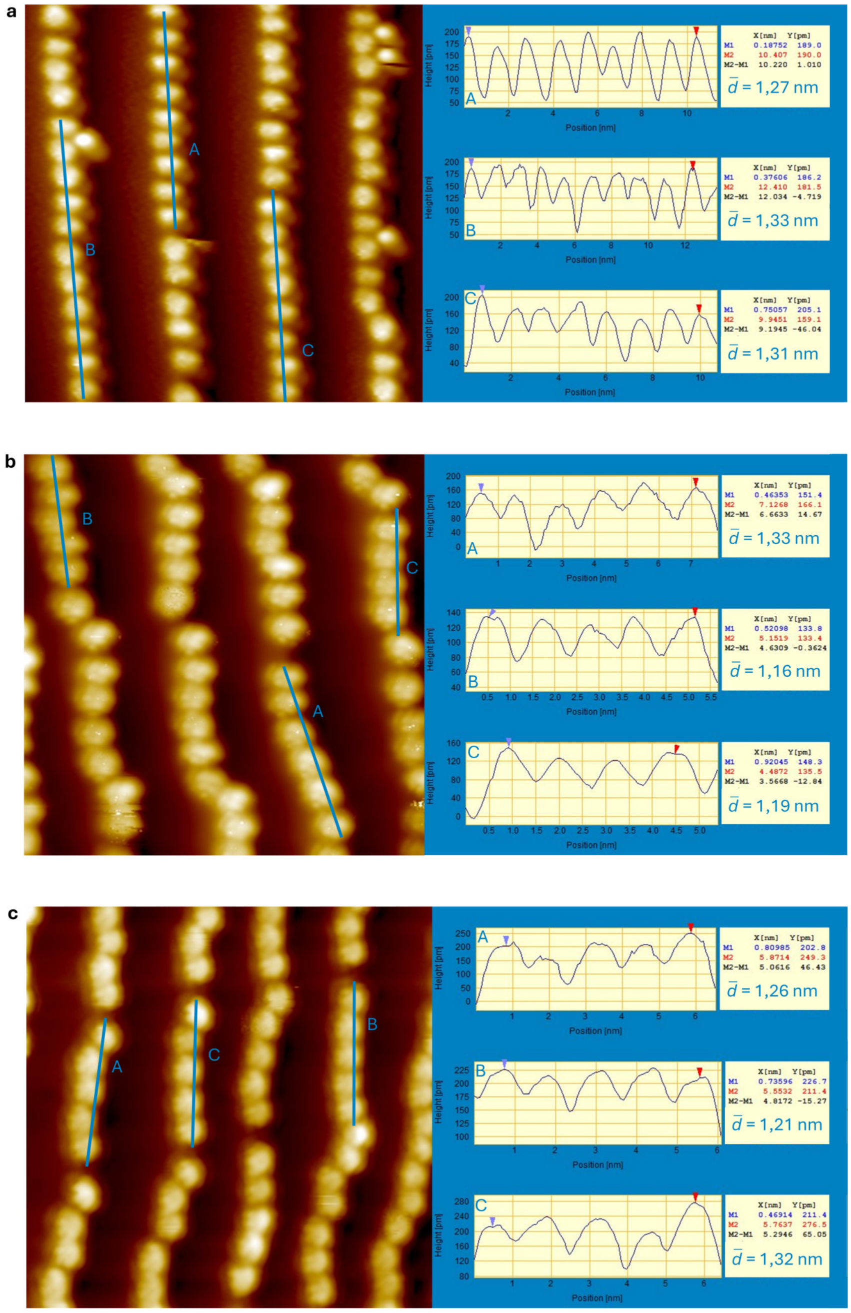


**Fig. S7 STM images for determining the average intermolecular distance of IPr.** For three different STM experiments (feedback 20 pA, 1 V) in **(a)**-**(c)** the intermolecular distance is determined. Within each measurement, three line scans along the NHC-functionalized step edge are evaluated.

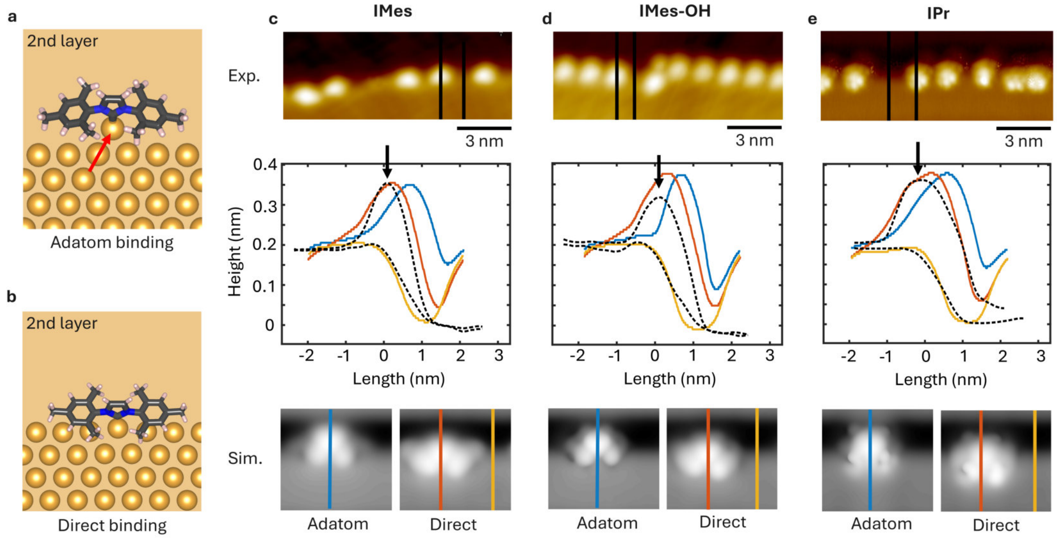


**Fig. S8 STM and DFT analysis regarding ad-atom mediated binding.** Two possible binding modes illustrated for IMes. **(a)** Ad-atom mediated binding. **(b)** The carbene is directly bonded to the step-edge. Experimental STM images (feedback 20 pA, 1 V, top), simulated STM images (bottom) and corresponding line scans (middle) for IMes **(c)**, IMes-OH **(d)** and IPr **(e)**. The blue line corresponds to ad-atom mediated binding, the orange line to covalent binding, the yellow line to the unfunctionalized step edge and the dashed black lines to the experiment.

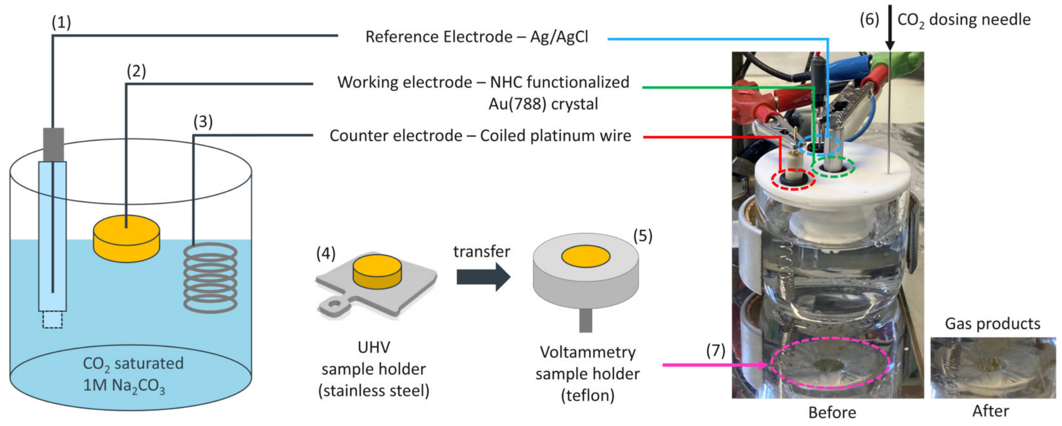


**Fig. S9 Detailed description of the electrochemical setup.** (1) Ag/AgCl reference electrode (RE). (2) NHC functionalized Au(788) crystal, used as working electrode (WE). (3) Coiled platinum wire, used as counter electrode (CE). (4) Sample holder used within the SPM and XPS UHV systems. (5) Voltammetry sample holder used in the electrochemical cell. Only the crystal surface is exposed to the electrolyte, while the backside is connected to the current collector. (6) The $CO_2$ dosing needle is inserted in the electrolyte and connected to the gas bottle. (7) The electrochemical cell is placed on a mirror to observe the crystal surface during the experiment. Bubbles occurring on the surface indicate that gas products are formed.

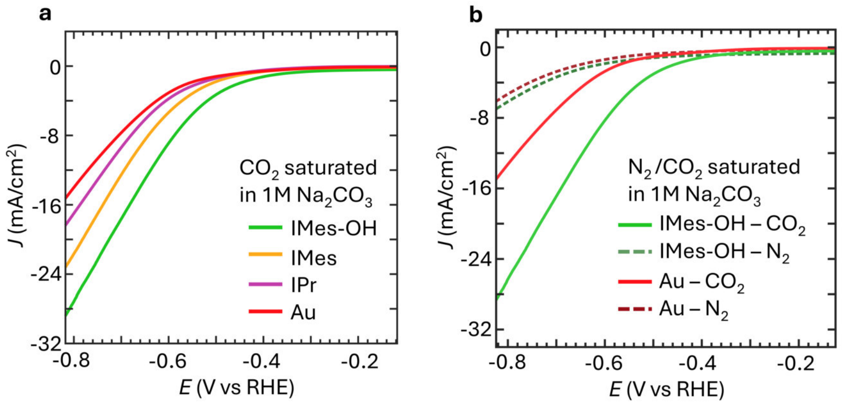


**Fig. S10 Catalytic performance in $CO_2$ and $N_2$-saturated electrolyte (a)** Linear sweep voltammetry curves in $CO_2$-saturated $Na_2CO_3$ electrolyte. **(b)** Linear sweep voltammetry curves in $N_2$-saturated $Na_2CO_3$ electrolyte.

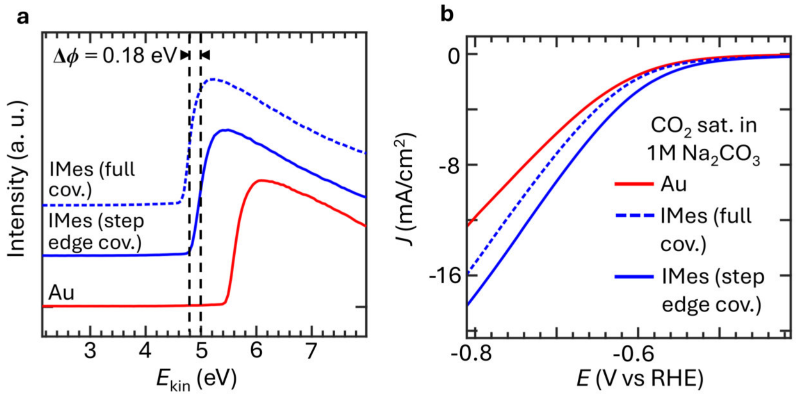


**Fig. S11 Coverage dependence of the catalytic performance and reduction of the work function for IMes. (a)** Secondary electron cutoff using XPS. For better visibility the curves are shifted vertically. **(b)** Linear sweep voltammetry curves for the electrochemical reduction of $CO_2$ to CO. The blue dashed lines correspond to measurements of a fully covered surface, whereas the blue solid lines correspond to a surface where only the step edges are functionalized. The red lines correspond to a measurement of the bare Au(788) surface. It should be noted that a different Au(788) single crystal was used for the measurements shown in (b) than for those shown in the main text (Figure 3). The polishing conditions from crystal to crystal, and consequently the density of step edges, may differ on a macroscopic scale. The data are largely comparable, however, the overall total current densities show slight variations.

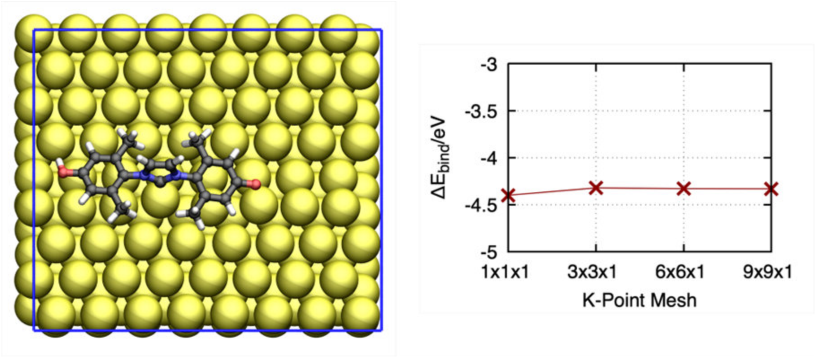


**Fig. S12 K-point mesh convergence analysis for IMes–OH on Au(788).** The left panel shows a top view of the supercell with the IMes–OH molecule adsorbed at the step edge of the Au(788) surface model. The right panel shows the calculated binding energy $\Delta E_{\text{bind}}$ as a function of k-point mesh density. The results demonstrate that the binding energy is already well converged using a $1 \times 1 \times 1$ mesh.

## References


[1] Redhead, P. A.: Thermal desorption of gases. *Vacuum* **12**(4), 203–211 (1962).

[2] Liu, L., Das, A., Wiesener, P., Tomut, A.-C., Tran, D., Das, M., Daniliuc, C. G., Fuchs, H., Doltsinis, N. L., Mönig, H., Glorius, F.: Hydrogen Bonding Networks Formed by Hydroxyl-Functionalized N-Heterocyclic Carbenes on Ag(111). *ChemRxiv* (0217) (2026). Preprint at https://chemrxiv.org/doi/full/10.26434/chemrxiv.15000173/v1.
